# Improving event-based methods for modelling flood risk in a variable and non-stationary climate

**DOI:** 10.1098/rsta.2024.0292

**Published:** 2025-07-31

**Authors:** Rory Nathan

**Affiliations:** ^1^ Department of Infrastructure Engineering, The University of Melbourne, Melbourne, Victoria, Australia

**Keywords:** event-based flood modelling, climate change, aleatory uncertainty, epistemic uncertainty, Monte Carlo analysis

## Abstract

While it may be easy to simulate the rise and fall of a flood hydrograph, it is surprisingly challenging to reproduce the magnitude–frequency relationship of a selected flood attribute (i.e. a flood frequency curve) under historic or current conditions, particularly over the range of spatial and temporal scales relevant to flood behaviour at different locations within a single catchment. Under climate change, these challenges only get harder. This article discusses the key aspects of flood behaviour that need to be considered when deriving magnitude–frequency relationships in a non-stationary climate, as commonly required by the industry for planning and design purposes. Specific attention is given to sources of (irreducible) uncertainty that are a function of natural variability, and those sources of (reducible) uncertainty that are due to data limitations and imperfect knowledge. Discussion is provided on the design information and tools that are required to shift practice from a reliance on deterministic models to stochastic frameworks, and the hydroclimatic factors needed to accommodate the non-stationary effects of global warming. The article concludes with an outline of additional investigations needed to support the development and application of enhanced methods, and includes commentary on the disconnect between hydrologic practice and academic research.

This article is part of the Royal Society Science+ meeting issue ‘Hydrology in the 21st century: challenges in science, to policy and practice’.

## 1. Introduction

Flood events have had devastating effects on communities around the world, and over the past few decades, their frequency has increased significantly compared to other natural disasters [1]. Indeed, the number of flood disasters over the decade ending in 2020 was more than twice the number that occurred in the preceding decade, representing 44% of all natural disasters over that period and affecting 1.6 billion people worldwide, the highest figure for any disaster type [2]. Assessing the risk of such floods is a complex but crucial task that is routinely undertaken by hydrologic practitioners for a wide range of stakeholders in the public and private sectors. Flood risk assessments are commonly required for many planning and design purposes, including the identification of urban and rural areas vulnerable to flooding, the design of structures and the development of mitigation measures, environmental protection and disaster preparedness and response.

Traditional flood estimation procedures have generally assumed that the hydroclimatic factors governing the assessment of flood risk are invariant with time [3]; however, there is now strong evidence that anthropogenic warming is changing flood drivers and that it is necessary to take this into account when assessing flood risks [1,4]. It is to be noted that the term ‘flood risk’ is used here to denote the governing relationship between the magnitude of a particular flood attribute (e.g. peak flow, volume or level) and its exceedance probability, as commonly used in engineering practice. The general effects of climate change on extreme events and floods are well summarized in the latest assessment by the Intergovernmental Panel on Climate Change [5]. There is clear evidence that the frequency and intensity of heavy rainfalls have increased and the mix of storm mechanisms are changing [6], where the rates of increases in short duration rainfalls (less than or equal to 1 h) are approximately twice the Clausius–Clapeyron rate of approximately 7% K^−1^ found for long duration rainfalls (greater than or equal to 24 h) [4,7,8]. Increases approximately consistent with the Clausius–Clapeyron relationship also apply to estimates of probable maximum precipitation [9,10]. There is some evidence that warming temperatures are causing precipitation events to become more front-loaded (i.e. an increasing proportion of rainfalls occurs within the first half of the event) across a wide range of event durations and intensities [11]. Global warming is also affecting antecedent soil moisture [12–15] and this affects flood seasonality [16] and magnitude [16–18]. In regions subject to snowfall, warmer temperatures at low elevations deplete snowpack depths that reduce the frequency of rain-on-snow events, but at higher elevations, their frequency is increased due to higher rainfalls [19]. Urban catchments are less sensitive to changes in soil moisture compared with rural areas, and given the small contributing areas and concomitant sensitivity to short duration rainfalls, the effects of global warming on floods are particularly pronounced in urban catchments [20,21]. Coastal floods arising from the compound interaction between extreme sea levels and intense rainfalls will also increase [6].

Given the overwhelming evidence that flooding in the Anthropocene will become increasingly severe, it is not surprising that Razavi *et al*. [1] argue for ‘new scientific paradigms and tools that capture the profound uncertainty and complexity surrounding climate, environment and societal futures,’ where they conclude that we ‘require a change in research and practice to embrace uncertainty’. While the case for change is clear, exactly how we might proceed is not. Here, I provide some general principles to guide the development of new paradigms and tools, and this includes a discussion on some key deficiencies in current event-based methods that should be addressed to better account for climate variability and change. I finish with some comments on additional investigations that would be required to improve the defensibility of our estimates, and this includes commentary on the disconnect between hydrologic practice and academic research.

## 2. Efficacy of current approaches

The different approaches used to undertake flood risk assessments can be divided into three broad categories, namely, those based on the statistical analysis of flood data (flood frequency analyses) and those based on the transformation of rainfall into streamflow using models that are employed either on an event basis or as a continuous time series. There are many variants of methods in each of these broad groupings, including hybrid approaches that combine elements from different categories. Somewhat surprisingly, there is very little published literature on the relative performance of different categories of approaches using design information available to practitioners, and this is a fundamental blind spot in our research agenda that is posited in §5. Some brief comments are provided below on the suitability of candidate approaches for estimating non-stationary flood risks. These comments are necessarily subjective and rest heavily upon my industry and research experience acquired over a 40-year period; the extent to which they relate to a specific design context will vary with data availability and design objectives.

Flood frequency analysis is thoroughly covered in the scientific literature and involves the fitting of a probability distribution to historical streamflow maxima. With sufficient data, the approach provides highly valuable information on flood risk, though in many regions, suitable data are either not available or not representative of the conditions of interest. Estimates of flood risk based on site-specific data are generally limited to annual exceedance probabilities (AEPs) up to approximately 1 in 100 (or 1%), which is relevant to routine drainage design and floodplain developments, but not to the design or assessment of infrastructure of national or critical importance [22]. The approach is not well suited to exploring mitigation options in constructed environments (i.e. problems sensitive to both the peak and the volume of flood loading) or in catchments subject to land-use change. Further, while covariates can be used to inform the projection of flood risk estimates [23,24] the choice of non-stationary model and the treatment of covariates have a large influence on projections [25] and represent a problem of ‘double extrapolation’ to magnitudes larger than found in the historic record and to warmer climates [26].

Continuous simulation employs hydrological models to simulate streamflow at sub-daily (or daily) time steps, and flood quantiles are then derived from the simulated streamflows using a suitable probability model [27]. These models are well suited to the analysis of distributed systems as the method implicitly accounts for the joint probabilities between the state variables involved, where catchment wetness antecedent to rainfall events is simulated using a suitable soil moisture accounting scheme. There are several disadvantages to the method, including the challenge of stochastically generating sequences of rainfall data that capture the required dependencies over a range of spatio-temporal scales [28], and there are significant technical difficulties involved in estimating flood risk at ungauged locations within or external to the model domain. In general, specialist skills are required to configure and calibrate the models, and in the context of climate change, there are difficulties involved in deriving rainfall time series that correctly reflect the different rates of change over different temporal scales [29]. This is a particular challenge when focused on the severe floods of concern, as these models are more highly parametrized and more vulnerable to problems of equifinality than event-based models, and there is little information available to characterize the flood response (e.g. even with 50 years of good quality records, there might be only three or four flood events of interest). Overall, continuous simulation models are well suited to solving a small proportion of practical problems involving distributed storage elements, but in many cases they introduce additional problems that are more easily solved by simpler methods.

Event-based modelling is the method most commonly used by industry practitioners. Some indication of how widely the approach is adopted can be seen in the list of jurisdictional guidelines that employ intensity–duration–frequency (IDF) curves (i.e. the cumulative density function of rainfall depths over a specified duration), as summarized by Wasko *et al*. [3]. At its simplest, the method involves creating a ‘design storm’ comprising a rainfall depth sampled from an IDF curve for a specific duration, which is averaged or distributed in time and space. This rainfall depth is transformed into a flood peak (or volume) using a runoff production model (to account for catchment losses), perhaps the most well-known examples of this being the ‘rational method’ developed by T. J. Mulvaney in 1851 [30] and the Soil Conservation Service curve number method [31]. Since the 1970s, it has been common to combine estimates of surface runoff with a hydrograph formation model (to account for overland and channel routing) to provide estimates of the full flood hydrograph, for which many linear and nonlinear models have been developed and applied in either a lumped or semi-distributed fashion (e.g. [32]). One key benefit of event-based approaches is that, with the right selection of inputs, very simple conceptual models can be used to accurately simulate the flood response of a catchment, particularly those that are not heavily urbanized and influenced by anthropogenic hydraulic controls. Another benefit is that event-based models can take direct advantage of rainfall records, which are more easily regionalized and hence extrapolated than flood records, and they can also transform regionalized estimates of probable maximum precipitation [33] into corresponding flood estimates for the design of critical infrastructure such as dams. Disadvantages of the approach include the need to estimate the state of catchment wetness prior to rainfall events, and the generally simplistic manner in which the temporal and spatial patterns of rainfall are represented. Perhaps the key weakness of the approach is that while the models might be able to simulate a plausible flood hydrograph, they are not well suited to characterizing the exceedance probability of an event. This is because the models are generally implemented in a simple deterministic manner, where it is commonly (and optimistically) assumed that careful selection of design parameters will ensure that the exceedance probability of the derived flood is the same as its causative rainfall.

Given the simplicity of event-based models, it is not surprising that they have been adopted so widely, though it is perhaps equally surprising that there is such a paucity of literature on their efficacy. One comparative error analysis [34] reported that flood frequency methods had better skill than simple event-based models, noting that the relative benefits of these approaches depend on the location of interest, the data available and the design objective of interest. The study by Kuczera *et al.* [34] is noteworthy in that the error analysis was applied to three methods as they would be undertaken using a specific set of (Australian) guidelines, and this approach is essential if we are to understand the relative benefits of such methods for practical design applications in a particular jurisdiction.

The remainder of the article focuses on the need and opportunities for the improvement of event-based models. This focus is adopted partly out of the practical need to limit discussion to a single manuscript, but, as discussed, this is also because they are the most commonly used design approach and they are well suited to incorporating the effects of climate change.

## 3. Inductive reasoning example

There are a wide variety of event-based approaches, yet there are some generic assumptions that are common to all such methods that can be critically reviewed using inductive reasoning. To this end, the next two sections explore some general limitations of event-based models, and this is followed by a discussion of how these might be overcome so that the methods are better suited to future challenges.

The application of an event-based model to flood risk assessment is illustrated using data from the Delatite River catchment located in southeastern Australia. This catchment was selected to provide an example for inductive reasoning, and it has no special significance other than the fact that the relevant data were readily available. A gauging station on the river (gauge 405 214) provides approximately 67 complete years of streamflow observations since March 1957, with continuously recorded rainfall observations available since November 2001; daily rainfall data averaged across the catchment were obtained from a gridded rainfall surface fitted to observed data [35]. The headwaters of this river rise in a mountainous region covered by forest with an annual rainfall of approximately 1400 mm, and flow through lower areas that have been cleared for grazing and agriculture with an annual rainfall of approximately 850 mm. The catchment area above the streamflow gauge is 368 km^2^, and the river descends approximately 1200 m over a distance of 85 km.

As described in detail below, the two key steps involved in this application are: (i) the fitting of a suitable hydrologic model to a sample of historic events to check that the model configuration and routing parameters represent the flood response of the catchment and (ii) the use of the model with appropriate design inputs to derive flood quantiles, and these are compared to independent estimates obtained from flood frequency analysis.

### (a) Simulating flood response

**Figure 1.**
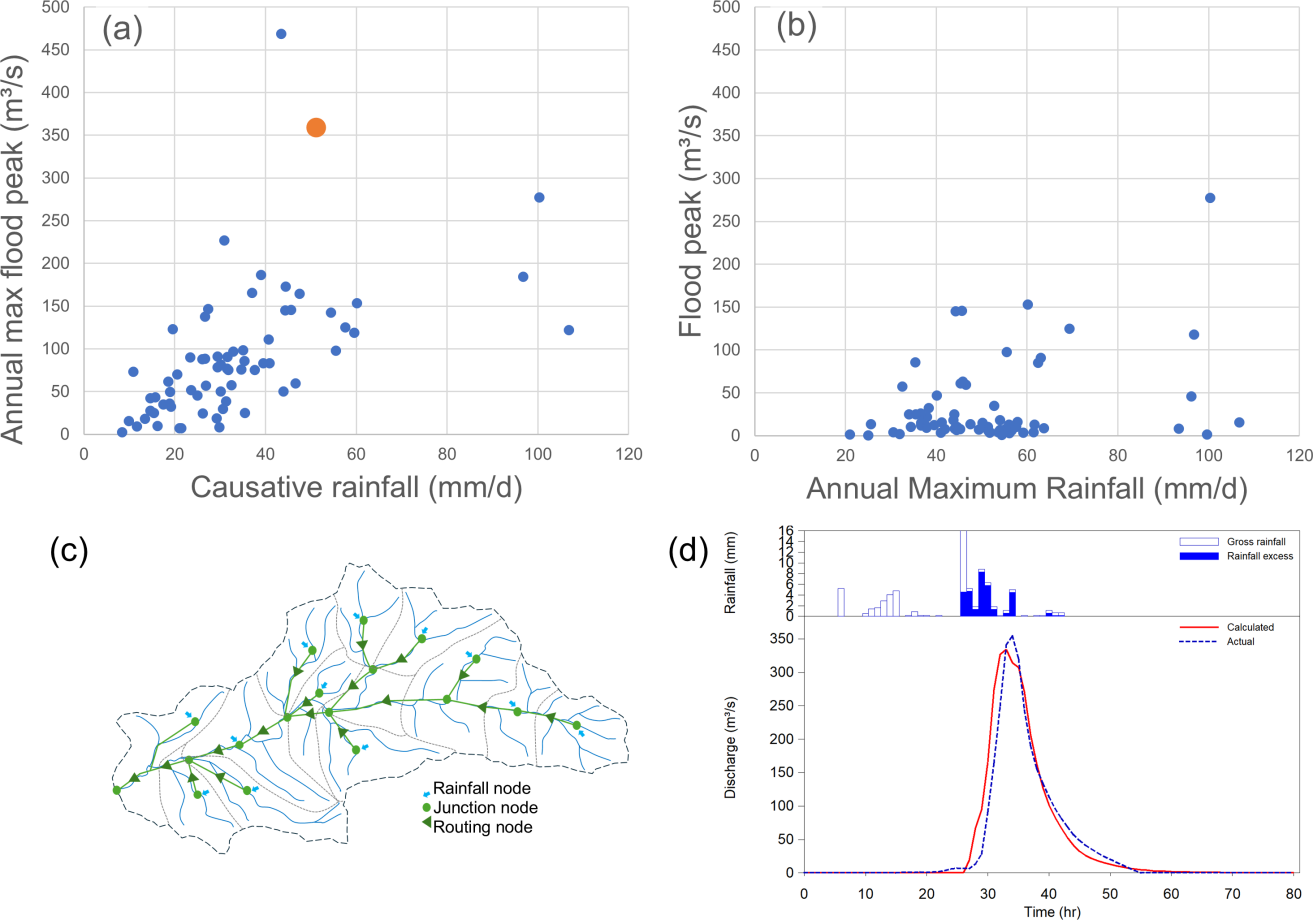
Scatter plot between daily rainfall and flood peaks in the Delatite River catchment when events are sampled on (a) maximum floods and (b) maximum rainfalls, including (c) a schematic of hydrologic model and (d) example comparison between simulated and observed flood peak for the second highest ranked event (orange circle in panel (a)).

Early on in a flood study, it is usual to search historic records to find information on the largest floods that have occurred in the catchment. The sample of available annual maximum floods and their causative rainfalls for the Delatite River is shown in figure 1*a*. A suitable flood model is configured to the catchment (figure 1c) and this is then calibrated using concurrent information on rainfalls to simulate the rise and fall of the flood hydrograph (as shown in figure 1d, which is based on the simulation of the second highest observed flood event, indicated by an orange symbol in figure 1a). There are numerous candidate models for this purpose. Traditionally, unit hydrograph models have been used to emulate the linear response of a catchment to rainfalls in a lumped fashion (e.g. [36]), and these have been combined with a suitable node-link routing model to represent nonlinear flood response in a semi-distributed fashion (e.g. [37]). In Australia, storage-routing methods are commonly used, where the RORB model is the first and most widely implemented method [38]. Details of the different candidate models and their calibration can be found in the relevant user manuals of the different models. For the purposes of the example presented here, it is reasonable to assume that the difference between candidate models is not of great significance, at least when applied by experienced practitioners to events found in the observed records (noting that different models will behave differently under extrapolation, which is a point discussed later). In other words, there are many candidate hydrologic models to choose from, and as long as appropriate care is taken to use good quality data, it is quite straightforward to simulate the flood response of a catchment using models with only three or four parameters.

Note that, while calibration events selected from a sample of observed large flood maxima are well suited to identifying model routing parameters, they are not well suited to identifying representative loss parameters. The reason for this is that flood events selected on the basis of their magnitude represent a biased sample, as large floods are more likely to occur when rainfalls are large and antecedent conditions are wet. This is an important point as the distribution of floods derived on the basis of large rainfalls (figure 1b) is quite different to those sampled on flood magnitude alone (figure 1a). Thus, to derive floods using probabilistic estimates of rainfall (i.e. IDF curves), we must take proper account of the joint probabilities involved by giving explicit account to the joint interactions between the major flood-producing factors.

### (b) Accounting for aleatory uncertainty

If floods were solely the result of large rainfalls, then the distribution of flood maxima shown in figure 1a,b would be identical. The differences between these two samples of flood maxima highlight the moderating influence of factors other than rainfall, and if we are to estimate the exceedance probability of floods using rainfall inputs (i.e. IDF curves), then we also need to account for the conditional probabilities involved in how rainfalls are transformed into flood peaks. The transformation of rainfalls into floods is inherently the result of a combination of factors, each of which is subject to aleatory uncertainty. The term ‘aleatory uncertainty’ is used here to refer to the inherent randomness or variability in factors like rainfall intensity, its temporal and spatial distribution and soil moisture that together result in a flood. This source of uncertainty is a fundamental characteristic of flood events that cannot be reduced through improved knowledge or data.

To use an event-based model with rainfall depths sampled from an IDF curve, it is necessary to provide an estimate of antecedent catchment wetness through the parametrization of a suitable loss model. Such losses represent the average depth of rainfall across the catchment that infiltrates into the ground or is lost to various interception and depression stores. It is also necessary to specify how the rainfall depth is distributed over the duration of the event, a function variously referred to as a temporal profile, or temporal pattern. In some catchments, it may be necessary to specify both the temporal and spatial distribution of rainfalls, an attribute that is referred to as spatio-temporal profile, or pattern. The difficulty is that antecedent conditions and (spatio-)temporal profiles are subject to aleatory uncertainty, but in practice, it is generally assumed that such factors are of minor importance and that it is sufficient to treat these as fixed values (e.g. the initial moisture content and temporal profiles recommended in [36] are not varied in a stochastic manner).

**Figure 2.**
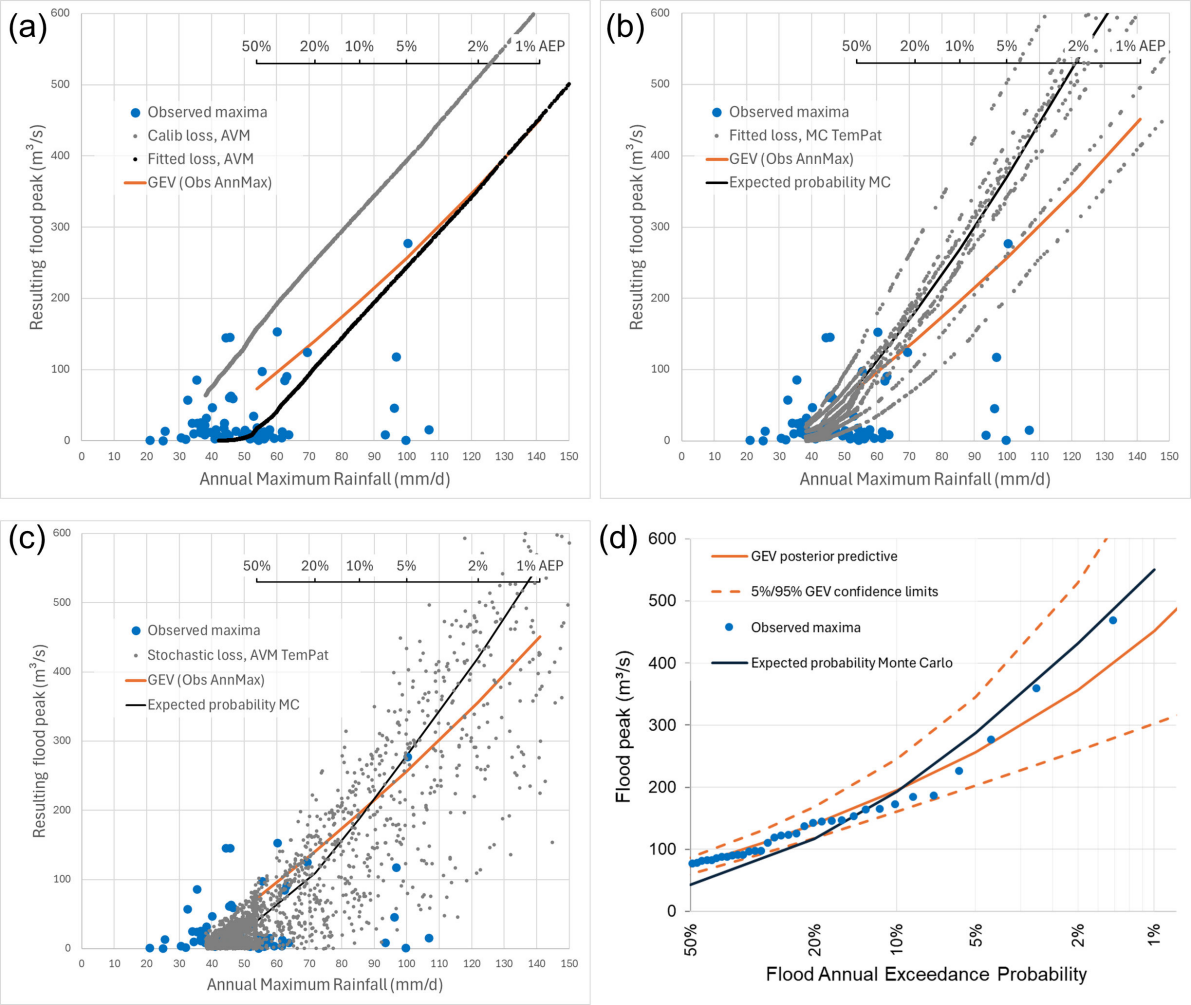
Ability of an event-based model configured for the Delatite River catchment to simulate aleatory uncertainty in flood peaks (grey circles and black lines) compared with real-world data (blue circles), compared with quantiles derived from flood frequency analysis (orange lines) using (a) fixed losses and a single temporal profile, (b) fixed losses and an ensemble of temporal profiles and (c, d) stochastic sampling of losses and temporal profiles. In panels (a–c), the peak flows are shown as a function of their causative rainfalls, and panel (d) shows the derived flood frequency curve.

A series of simulations is presented below that highlight the consequences of progressively introducing aleatory uncertainty into the simulations. Aleatory uncertainty in rainfalls alone is first considered, and then this is followed by the progressive consideration of aleatory uncertainty in temporal patterns, then losses, then a combination of all three factors combined (for simplicity, here, it is assumed that the spatial distribution of rainfalls has a minor effect on flood magnitude and a single fixed spatial is adopted for all simulations). The influence of these sources of aleatory uncertainty is investigated using Monte Carlo simulation, whereby rainfalls, losses and temporal profiles are selectively sampled from their governing probability distributions, and expected probability estimates of the resulting flood quantiles are derived using the total probability theorem [39,40]. To guide this investigation, an independent estimate of the ‘true’ distribution of flood maxima is obtained from the posterior predictive distribution obtained by fitting a general extreme value distribution in a Bayesian analysis [41] (these estimates are also known as ‘expected probability’ quantiles and represent the average probability of an extreme event being exceeded). These independent estimates of the true distribution of flood maxima as a function of rainfall are shown as a solid orange line in figure 2a, where the observed flood maxima used to derive the estimate are shown as solid blue circles (this is the same sample of maxima as shown in figure 1b). Also shown in figure 2a is a scale showing the AEP of the rainfall depths as determined from the relevant IDF information.

First, to consider aleatory uncertainty in rainfalls alone, it is common industry practice to adopt a fixed loss parameter representative of average soil moisture deficit across the catchment, and a single temporal profile representative of the typical distribution of rainfall rates over the duration of a storm. A variety of approaches have been adopted to characterize such ‘representative’ values of losses (e.g. [36,42]) and temporal profiles (e.g. [43]; vol 2, section 4.2 of [44]). The results obtained by adopting loss parameters that are based on average values obtained by fitting a flood model to historic events, and a temporal profile based on the average variability of patterns found in a sample of 10 regional storms [43], are shown by the line of grey points in figure 2a. The points shown in this figure represent the variability in flood response due to aleatory uncertainty in rainfall depth only (achieved by sampling from the IDF curve), where all the derived floods lie on a single line as antecedent catchment wetness and temporal profiles are held constant. The other obvious point to note here is that these results lie well above our independent estimate of the true relationship between rainfall and floods, as represented by the Generalised Extreme Value (GEV) distribution fitted to observed flood maxima (orange curve, figure 2a).

As discussed earlier, it is not surprising that the use of average loss parameters obtained from calibration to historic events lies above the true value as they are based on a sample of events that are biased towards wet conditions (i.e. losses are biased low). The traditional correction for this problem is to adjust the losses within a reasonable range to achieve better agreement with the estimates based on flood frequency analysis [44,45, vol 2, sec 4.1]. A trial-and-error approach can be taken manually given that the loss models used in such applications are very parsimonious. In this example, it is found that increasing the continuing loss rate from 1 to 2.5 mm h^−1^ yields the line of black points shown in figure 2a, which is in reasonable agreement with the more extreme events (i.e. 5% AEP and rarer) as determined by flood frequency analysis. With this simple change in the continuing loss rate, we now have an event-based model that reproduces the relationship between flood peak and rainfalls in a manner that reasonably preserves the exceedance probabilities involved. Of course, the problem with this approach is that it ignores all the joint interactions between antecedent conditions, temporal patterns and rainfall depths, and thus is unable to reproduce the aleatory uncertainty inherent in flood processes evident in real-world data (blue circles, figure 2a). Without the independently derived flood frequency quantiles for comparison, it would not be possible to know how best to adjust the loss values. In essence, this approach is an exercise in ‘curve fitting,’ whereby all deficiencies in the ability of the model to simulate flood processes are attributed solely to errors in representing average catchment losses. It ignores, for instance, the (often substantial) catchment-specific effects of storm temporal profile on flood response due to differences in channel network topologies and source areas. For example, while the initial soil moisture parameters in the ReFH2 event-based method have been calibrated to match the 2 year return period peak flows, these values are further adjusted to take account of changing event severity; the use of such an adjustment factor is recognized as being ‘a pragmatic solution that is unattractive from a hydrologic perspective’ and thus the ReFH model estimates cannot ‘be regarded as being independent of the statistical method’ [36, p. 6]).

Next, to examine the influence of aleatory uncertainty on temporal profiles, the adoption of a single fixed representative temporal profile is replaced by the stochastic sampling of an ensemble of temporal profiles derived from large storms observed in the region[46]. These storms are the same set of storms used to derive the single temporal profile of average variability used in the previous example. The results for this simulation are shown in figure 2b, where it is now seen that each temporal profile yields its own separate relationship between rainfall and flood peak. This is seen to capture some of the aleatory uncertainty inherent in the real-world data, but the relationships are still heavily deterministic and clearly unlike the observed patterns of natural variability. Interestingly, when expected probability quantiles are computed based on these 10 temporal patterns (solid black line, figure 2b) it is seen that the losses derived assuming a single representative temporal profile (solid black line, figure 2a) now yield results that are inconsistent with those based on flood frequency analysis (orange curve, figure 2b).

To now consider a more complete representation of aleatory uncertainty, the foregoing simulations are repeated, but this time losses are now allowed to stochastically vary in a manner consistent with observed behaviour [42]. The simulations now represent the combined uncertainty arising from variation in all three major flood factors (rainfall depths, temporal profiles and losses), and it is seen (figure 2c) that the model now captures the aleatory uncertainty in floods for all events rarer than approximately 50% (i.e. 1 in 2) AEP. It is clear that if we are to simulate these very frequent floods, then we need to consider aleatory uncertainty in other factors, and while this might be explained by considering stochastic variability in the spatial patterns of these inputs, there is little motivation for doing this, given the focus is on more extreme events.

The results of the foregoing simulations are shown in terms of the scattered relationship between rainfalls and peak flows because this best illustrates the underlying processes of most interest, but it is also useful to examine the results in the probability domain, as ultimately it is the relationship between flood peak and exceedance probability that is most useful for design and planning purposes. Accordingly, the expected probability quantiles derived from the Monte Carlo simulation shown in figure 2c (black line) are re-plotted in the more familiar probability domain in figure 2d alongside the flood frequency results obtained by fitting the GEV distribution to the observed annual maxima floods. It is seen that the flood quantiles derived from the event-based modelling lie well within the 5%/95% confidence limits of the fitted GEV distribution (except for the more frequent events), which indicates a reasonable level of agreement between both sets of estimates.

### (c) Non-stationarity and epistemic uncertainty

The preceding discussion illustrates how the use of a simple event-based model in an appropriate stochastic simulation framework is able to represent natural variability of flood processes, and when suitably parametrized is able to reproduce the frequency of floods over a range of event magnitudes. This ability provides confidence that we are not merely engaged in a curve-fitting exercise in which the parameters of a parsimonious model are adjusted without regard to physical reasonableness, an approach that risks achieving the right result for the wrong reasons. While curve-fitting might provide a defensible approach under stationary conditions when the estimates of interest lie within the range of observations, it is not suited to extrapolation outside the observed range, particularly in a non-stationary environment where the frequency and/or intensity of some flood-producing factors might change more than others. As discussed in §1, climate change is affecting a range of flood-producing factors. Unless such changes are explicitly and separately represented, we cannot expect to have confidence in our ability to simulate the conditional joint probabilities involved, and hence we cannot expect to correctly estimate the changes in the derived flood frequency curves either under current or future conditions.

**Figure 3.**
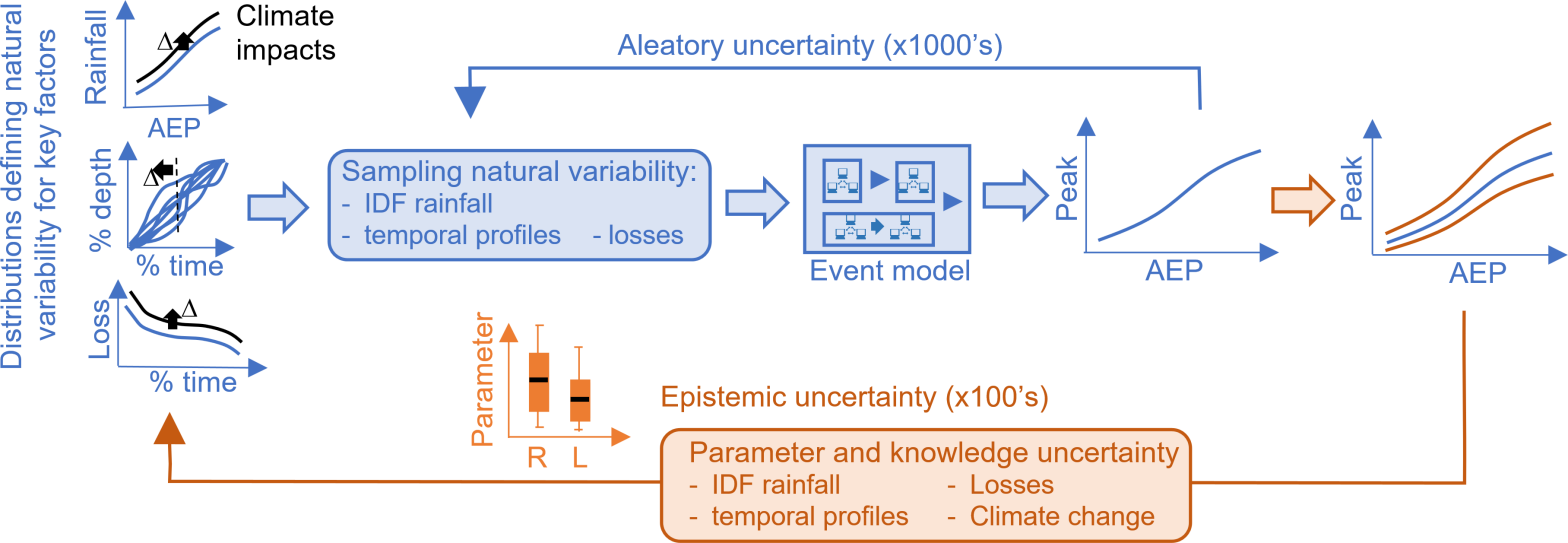
Illustration of simple event-based modelling scheme in Monte Carlo simulation framework showing inner-loop simulation of aleatory uncertainty in key flood-producing factors to derive best estimate of flood risk (blue) and outer-loop simulation of associated epistemic uncertainties to derive confidence limits (orange), where adjustments made to the inputs to represent non-stationarity are shown in black.

A high-level schematic of an approach suited to representing the different sources of uncertainty is shown in figure 3. This figure represents the simulation scheme comprising two main computational loops. The inner calculation loop (coloured blue) is used to sample all the possible joint interactions between different sources of aleatory uncertainty, where each completed set of simulations yields a derived flood frequency curve that ignores the uncertainties due to data limitations and knowledge about processes. The outer loop (coloured orange) is used to characterize epistemic sources of uncertainty. The term ‘epistemic uncertainty’ is used here to refer to the uncertainty that arises from our limitations in knowledge, data and understanding of the underlying physical processes. It is a type of uncertainty that can potentially be reduced through improved information and modelling. In essence, if only a best estimate is required, then only those simulations (in blue) need be undertaken and the uncertainty around this best estimate is ignored. If we wish to understand the uncertainty around this best estimate due to knowledge or data limitations, then we also need to undertake the simulations shown in orange. Some additional details on these two computational loops are provided below.

For the inner loop, it was shown earlier that a best estimate of the flood frequency curve can be derived by stochastic sampling from the parametric (or non-parametric) distributions that govern the natural variability in the key flood-producing factors. These stochastic combinations of inputs are used to force the event-based model, and a statistical analysis is then undertaken to derive a best estimate of the derived flood frequency curve [39,40]. Of course, the challenge under non-stationary conditions is that our best understanding of the changes to the marginal distributions that govern flood behaviour is based on a body of literature that considers both trends in the historic record and projections obtained from detailed climate (and sometimes hydroclimatic) modelling over a range of spatial and temporal scales. Such understanding is generally not available for the catchment of interest, but rather needs to be inferred from multiple lines of evidence obtained from global and regional analyses. The problem facing the industry is that the nature of the evidence available on climate effects is not directly applicable to the procedures used for design flood estimation. There are three aspects to this, namely:

— first, the spatial and temporal scales of most relevance to drainage design and floodplain management are often one or two orders of magnitude finer than the evidence for climate effects presented in the scientific literature (noting that regional climate models with convection-permitting schemes can reduce this disparity [47]);
— second, it is often not clear how to relate projected climate change effects to their conceptual equivalents in the different procedures (e.g. quantitative metrics on drying soils do not directly relate to averaged loss parameters used in event-based models); and
— third, the methods used by industry are often based on a broad legacy of data sources collected over a long period of time, and it is difficult to identify what baseline periods are relevant to different elements of the design data compendium; as such, it is not straightforward to determine how best to incorporate evidence for non-stationarity as it becomes available.

**Figure 4.**
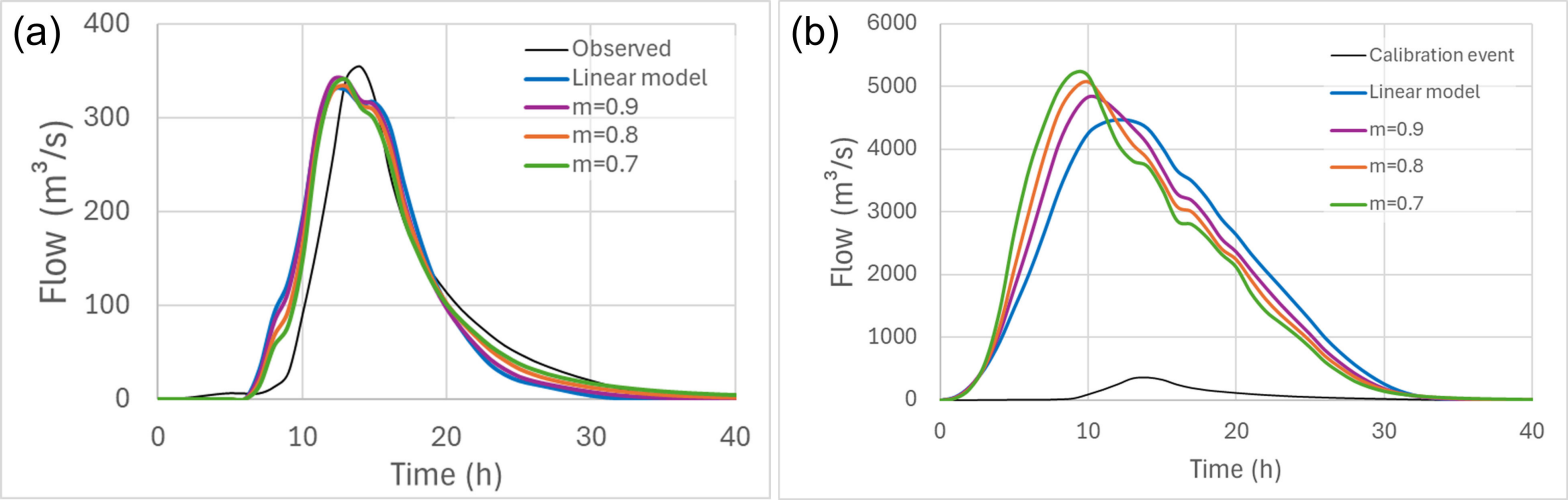
Illustration of (a) the ability of different models to simulate historic event equally well, and (b) the same models under extrapolation when used to estimate the PMF in the Delatite River catchment.

While this problem is tractable (with sufficient resources), event-based models are perhaps more amenable to the incorporation of climate change considerations than other design procedures. In concept, the mechanics of representing the effects of climate change in event-based schemes is straightforward, as illustrated by the adjustments shown in black in figure 4. The required perturbations can be linked directly to temperature changes or specific climatic scenarios, where scaling factors relevant to changes in rainfall intensity [7]—which is generally the dominant flood driver—are applied to existing design rainfall (IDF) inputs. Information on how changes in antecedent conditions affect design loss parameters can be derived from hydroclimatic projections [15] and then made available to practitioners in the form of generic scaling factors similar to rainfall. Any information on how temporal profiles may change [11] can be used to perturb the profiles directly or else be used to inform a changed sampling strategy. The point of the examples provided here is not to present a specific solution, but rather to illustrate how information obtained from multiple lines of published evidence can be regionalized and be directly incorporated into existing design procedures [29,48]. In contrast, the process of adjusting estimates of flood frequency quantiles for climate change effects [23] is more complex and less easily regionalized, and accounting for the complexities of climate effects on evapotranspiration demands and rainfalls over multiple scales for continuous simulation presents additional challenges for scientists and practitioners alike [49].

The outer loop (orange-coloured elements of figure 3) is used to represent the epistemic sources of uncertainty associated with data limitations (e.g. gauge locations, rating curve errors and lengths of record), parametrization (and structure) of the modelling chain components and in the scaling factors (and assumptions) involved in accounting for climate change. The effect of these uncertainties can be assessed through a simple sensitivity analysis by merely investigating which inputs or model components have the largest effect on the results; more rigorously, the simulations can be undertaken by stochastically sampling from the distributions that govern the uncertainty in the key parameters most affecting the results. The distribution of the outputs arising from the sampling of epistemic uncertainty thus provides uncertainty around the best estimate, as derived by solely considering aleatory sources of uncertainty.

Accounting for sources of epistemic uncertainty is rather more difficult than for aleatory sources. Data sources relevant to aleatory uncertainty are both abundant and readily accessible, and since they are associated with the behaviour of natural systems, they are amenable to regionalization. In contrast, characterizing epistemic uncertainty tends to be problem-specific, as the uncertainty of the estimates is heavily dependent on the model being used, the data available for calibration, and the degree of extrapolation required (spatially, temporally and in magnitude).

Perhaps the two most salient sources of epistemic uncertainty in event-based modelling are (i) the need to provide flood estimates for locations where there are no data and (ii) the parametrization and structure of both routing and loss models. With regard to the first source of uncertainty (ungauged locations), it might be expected that the relative ease of deriving IDF information using regional pooling methods provides a sound basis on which to derive site-specific flood estimates. However, such estimates also rely on the regionalization of parameters for the adopted flood model, and the resulting accuracy of the flood estimates has been found to be comparable to, but no better than, regional flood frequency estimates [34,50]. With regard to the parametrization and choice of routing model, a simple example illustrating the challenges involved is shown in figure 4. The left-hand panel of this figure shows the calibration performance of four different storage-routing event-based models [38], noting that the historic event selected is the second highest flood event in 68 years of systematically gauged observations (this is the same event that is shown in figure 1a,d). One storage-routing model is configured to provide a linear response to rainfall inputs, which is equivalent to the well-known unit hydrograph model as adopted in the UK ReFH approach [36]; the other three are nonlinear models, where the power exponent in the storage–discharge equation varies between 0.7 and 0.9, which represents a realistic range of behaviour around default industry guidance in Australia [51]. It is seen from figure 4a that the calibration performance of the nonlinear and linear catchment routing models is nearly identical, and for the point being made here, it can be assumed that all models are able to reproduce any selected observed flood characteristics similarly well, given the same set of inputs.

However, the ability to reproduce flood characteristics found in the historic record is a necessary but quite insufficient attribute, as the design objective of interest will invariably involve some degree of extrapolation, either in space or in severity; if this were not the case, then there would be little need for modelling. One common example of the need for extrapolation is the design and evaluation of critical infrastructure such as dams and nuclear power plants, as their performance is assessed against their ability to safely withstand the probable maximum flood (PMF). The PMF is a flood derived under upper-limiting assumptions to represent ‘maximum’ loading conditions [52,53], and such events are generally an order of magnitude larger (or two to three orders of magnitude rarer) than the largest events found in the observed record. When the four models used to calibrate the event shown in figure 4a are used to derive estimates of the PMF (figure 4b), it is seen that under extrapolation, the models yield demonstrably different results. While, in this case, there is approximately a 14% difference between the largest and smallest of the peaks, such differences will depend on the assumptions made and the characteristics of the specific catchment.

Evaluation of model performance under calibration using data of variable quality, or under extrapolation, could be strengthened by the use of formal statistical procedures [54,55], but the techniques involved remain in the specialistic research literature and would not be easily generalized for adoption by practitioners. There is considerable scope to inform such extrapolations using more physically based schemes, and given that many flood studies involve the use of two-dimensional hydraulic models these could be used to help configure the structure and/or parametrization of simple conceptual models, particularly in specific sections of the catchment where there are marked changes in conveyance associated with overbank flows and large floodplain storage. Alternatively, ‘virtual reality’ hydrological datasets can be prepared and used to assist the identification of model structure and parametrization [50,56], though of course any such approach needs to be consistent with physical reasoning and available evidence to provide confidence in the extrapolations of interest. The issue of which data to use and how they is interpreted is not without controversy (e.g. letter Dp in [57]). An example where observational data were used to identify the preferred structure of a loss model is provided by O’Shea *et al*. [58]. While this study provided evidence that a loss model needs to be independent of rainfall to perform well under extrapolation, considerable effort was expended on pooling and standardizing data across multiple sites to obtain a sufficient number of large events for evaluation, and this is the kind of analysis best undertaken in a research context.

## 4. Research gaps for industry application

The examples and discussion provided in the preceding sections provide some rationale for how industry practitioners could be better equipped to provide estimates of flood risk in a variable and non-stationary climate. There is a need to move away from deterministic procedures and transition to methods that treat the key flood-producing factors in a stochastic manner, where the joint probabilities involved and the effects of global warming are explicitly considered. Event-based models are well suited to making this transition; as they have very low computational overhead, they are able to take direct advantage of advances in our understanding of global warming, and the design inputs for models are easily regionalized. Thus, while in concept the transition from a deterministic to a stochastic framework represents a fundamental paradigm shift, the required datasets and tools can be developed by specialist providers and then made available to practitioners in a manner that facilitates their rapid adoption. The specifics of what is required will vary with design practice and thus by country (and possibly by jurisdiction), but the points below highlight some research topics that are generically applicable to event-based modelling.

*Non-stationary design rainfalls (IDFs*). There is unequivocal evidence that rainfall intensities are increasing with global warming, and as a consequence, many countries already provide for uplift factors to be applied to existing IDF relationships [3,59,60]. However, despite the large body of evidence suggesting that estimates of probable maximum precipitation need to be increased [4,10,61], to date it appears that only one country (Australia) has provided uplift factors for extreme rainfalls that are relevant to the design of critical infrastructure [48]. Quantifying how uplift factors may vary with storm duration, location and storm severity has important implications for design practice, but any improvements in our understanding of such influences can be readily incorporated into existing event-based models.

*Complete storms versus storm bursts*. Traditional design practice assumes that rainfall events occur in intense bursts of fixed duration, and any preceding rainfalls contiguous with the bursts are ignored (i.e. no account is given to rainfalls that would be considered to be part of the storm, but which fall outside the fixed durations). A proper discussion of this topic lies outside the scope of this article (though can be found in [60,61]), but the important point to note here is that the use of complete storms rather than bursts means that losses can be considered to be independent of rainfall and this greatly simplifies and improves the simulation of flood processes, particularly when extrapolating beyond conditions found in the historic record. Research is required on how best to characterize rainfalls that occur prior to bursts, whether they can be treated as deterministic or stochastic inputs, and how they might depend on event severity, antecedent climate-drivers and on global warming, noting that relevant information on this issue may be contained in the results of scaling studies based on complete storms (e.g. [62]).

*Temporal profile ensembles*. As illustrated in the inductive reasoning example, to simulate the processes leading to floods correctly, it is necessary to explicitly consider variability in temporal profiles. Information on temporal profiles can be readily obtained from observed records, but research is required to determine how best to regionalize and characterize suitable ensembles for use by practitioners with local design procedures. Such research should include investigation of the possible effects of global warming on temporal profiles [11], and possible dependencies of such effects on storm duration and location.

*Catchment wetness*. Variability in catchment wetness can have an appreciable effect on flood behaviour and thus also needs to be treated in a stochastic manner. It might be expected that variability in losses is catchment-specific and, unlike temporal profile ensembles, which can be easily regionalized, this might present an onerous change for practitioners. However, it has been shown that while the magnitude of losses may vary between different catchments, the distribution governing the likelihood that a particular catchment is wetter or drier than median conditions is similar across a wide range of hydroclimatic regimes [63,64]. This result holds for the simple initial loss/continuing loss model across the whole of Australia, and research is required to determine whether similar distributions can be derived for different loss models in different regions, and how typical losses are expected to change in a warmer climate [14,15]. If so, then practitioners need only estimate median loss parameters for their catchment of interest—which in concept is the same task that is required for deterministic modelling—the only difference being that with deterministic procedures this median loss parameter is held constant, whereas with stochastic sampling the median loss is the location parameter that allows the regional distribution to be anchored to the specific catchment of interest.

*Regionalized sources of epistemic uncertainty*. Providing information on sources of epistemic uncertainty for use by practitioners is perhaps the most difficult challenge. While it is straightforward to characterize epistemic uncertainty in rainfall quantiles for IDF relationships (a topic that is extensively covered in the scientific literature), it would not be straightforward to do this for other steps in the modelling chain that are more catchment-specific, such as routing model parameters governing the transformation of runoff into streamflow at a selected point in the catchment, and estimates of loss model parameters representative of typical catchment wetness. Some modelling frameworks provide capability for handling epistemic uncertainties [37], and in concept this could be extended to the consideration of uncertainty in model structure, but to be useful, practitioners also need information on the governing uncertainty distributions.

*Reconciliation with other lines of evidence*. Given the inherent uncertainties involved, it is important that processed information is available with which to evaluate the defensibility of a given flood estimate. All methods are subject to assumptions and limitations, and evaluating the extent to which different estimates can be reconciled by varying assumptions within reasonable limits provides an important means of deriving defensible estimates for design purposes. The reconciliation of flood estimates requires the development of processed datasets that can be readily accessed by practitioners. For example, the provision of regional information on how flood attributes change with upstream contributing area provides an independent means of testing model extrapolations to ungauged areas [65]. Also, sources of processed information on expected changes in peak flow under climate change [66] can be used to compare with corresponding estimates from rainfall-based design methods, and even if the design in question is dependent on flood volume as well as peak rate, such information is useful to test the defensibility of the model being used.

*Integration with hydraulic modelling*. In many applications, it is necessary to use hydraulic modelling to convert estimates of peak flows into flood levels, and it is generally not feasible to do this directly with detailed hydrodynamic models because of the onerous computational overhead involved when employed in a stochastic framework. There is some evidence that the effect of uncertainty on flood flows is rather greater than on flood levels [67], but the degree to which uncertainty is attenuated is likely to vary with catchment conditions. There are a variety of ways this can be done, ranging from the use of simpler one-dimensional models [67], simple spatially distributed models [68] and the use of mathematical transforms to provide high resolution and accurate flood predictions from low-fidelity models [69]. The best practical means of undertaking this in a manner that integrates with flood models represents a fertile area of ongoing research.

*Non-stationary design considerations*. Planning and design decisions have traditionally been based on ‘risk-based’ concepts such as the average periods between the occurrence of a flood exceeding a given threshold (‘return period’), or the annual probability that a flood of a given size is equalled or exceeded (AEP); design practice is also commonly based on ‘standards-based’ criteria, such as a specific maximum flood level (e.g. given freeboard above a maximum historic event), or the ability to safely withstand a PMF. Such criteria were developed when it was assumed that extreme maxima arise from a stationary regime, but under climate change, their interpretation and rationale are not easily defended. Techniques are available for assessing performance in a non-stationary environment [70,71] and it will be necessary to ensure that future modelling frameworks provide information in a manner that supports decision-making under such conditions.

## 5. Discussion

The foregoing explication focuses on some of the challenges involved in working with design-event procedures and specifically addresses the flawed assumption common to all such methods, namely, that the exceedance probability of the derived flood is the same as its causative rainfall. While the form of the inductive reasoning example used to highlight the limitations of this assumption may be novel, the point being made is not: many practitioners and scientists familiar with flood behaviour are aware of the confounding issues (e.g. [72,73]), and perhaps the only surprising point is to wonder why we have relied upon indefensible deterministic assumption for so long. It is worth noting that the examples discussed here focus on flood processes that are common to rural catchments; they do not cover other factors that can have an important influence on flood response, such as the changing seasonal dependence between storm rainfalls and antecedent conditions, and the need to explicitly consider aspects of urban catchments and the hydraulic controls that differentiate urban flood response from rural and peri-urban environments. The relative importance of such factors varies with local conditions, and unfortunately it is beyond the scope of this paper to discuss these issues here. Scientists who investigate flood behaviour may argue that continuous simulation techniques implicitly solve the joint probabilities involved, and thus may question why practitioners seem so wedded to event-based procedures. However, the counterargument from industry is that continuous simulation techniques are only suited to a specialist class of problems, and that their use more generally introduces a raft of other problems that are more elegantly solved by event-based approaches.

This difference of opinion points to an apparent disconnect between the tools scientists use to investigate and publish on flood behaviour, and the needs of practising hydrologists who need to solve ‘real-world’ problems within resource and financial constraints, subject to industry codes of practice (or guidelines) that govern their legal and professional obligations. Identifying the research gaps and changed practices required to advance the defensibility of flood risk assessments is thus somewhat of a challenge, as the scientists who are best equipped to undertake the research are unlikely to have the necessary practical skills and experience to understand the industry context relevant to the problem.

The apparent disconnect between research and practice is most evident when considering the non-stationary implications of climate change, where it appears that to date we have spent more effort on understanding the physical science governing the effects of global warming than we have on how best to tackle the problem of changing risks in Anthropocene flood systems. Some indicative evidence for this is provided in the number of papers published on different aspects of the problem, as obtained from the Scopus search engine when populating the article title, abstract and keywords fields. From row A of table 1 it is seen that a total of 22 912 papers have been published on the effects of climate change on floods, but only approximately 13% of these focused on the design and frequency aspects of the problem, as suggested by the inclusion of the additional terms listed in row B of the table. While flood frequency analyses provide the most direct way of characterizing the relationship between flood magnitude and exceedance probability, practitioners commonly rely on rainfall-based methods to assess the effects of proposed changes to infrastructure and land use, and to estimate inundation caused by a combination of both flood peak and volume. It is thus interesting that the search results listed in row C of table 1 suggest that only approximately 1% of papers investigating the effects of climate change have used rainfall-based techniques, and of these, only 53 papers (0.2% of the total) have investigated effects using event-based models. The papers covered by this high-level review are published in journals ranging from contributions to science and case studies for industry (see the electronic supplementary material), and generally exclude the ‘grey literature’ commonly used in industry to promote best practice. Of course, the quality and defensibility of all such papers vary markedly with journal selection and the governance arrangements used to publish grey literature.

**Table 1.** Indication of the differences in research effort given to evaluating the effect of climate change on floods using a broad range of techniques, and those evaluated using methods adopted by practitioners.

row	notional scope	Scopus search terms	number of results	proportion of effort (%)
A	investigation of climate change effects on floods	‘floods’ AND ‘climate change’	22 912	100
B	investigation of the effects of climate change on the magnitude and/or frequency of floods	as for row A, AND (‘design’ OR ‘return period’ OR ‘exceedance probability’)	3082	13
C	as for row B, but using rainfall-based techniques	as for row B, AND (‘continuous?simulation’ OR ‘event?based’ OR ‘rainfall?based’ OR ‘grid?based’ OR ‘hydrol* modelling’)	228	1.0
D	as for row B, but using rainfall-based techniques relevant to design practice	as for row C, but search fields (and paper content where needed) were checked for relevance to event-based modelling procedures	55	0.2

The information listed in table 1 illustrates the apparent disconnect between the science that underpins our understanding of the effects of climate change and the types of research questions that need to be considered if we are to tackle the challenge outlined by [1]. It needs to be stressed that this summary is only intended to be indicative of the different areas of research focus, where the key message is that we while have a good understanding of the broad effects of climate change on floods, relatively little effort has been spent on how best to incorporate such understanding into practical flood guidance. Further evidence for this disconnect is provided in [4], where it is noted that while the design flood guidance for Belgium, Denmark, England, New Zealand, Scotland, Sweden, UK and Wales all recommend the use of climate change adjustment factors for rainfall intensities, no guidance has been provided for adjusting rainfall sequences for continuous simulation approaches; this is somewhat curious as there are a certain class of distributed volume-dependent problems that are best solved by continuous simulation, and this is also the most common tool used in the scientific literature. Other examples where it might be expected that there is a body of published work available to support the consideration of non-stationarity in practical guidance includes: operational estimates of probable maximum precipitation (as commonly required for the design of such critical infrastructure as nuclear power plants and high-hazard dams), changes in soil moisture (and hence catchment wetness antecedent to floods) and flood frequency analysis [74]. Note that, interim guidance for non-stationary flood frequency analysis is available in the UK [23], and that guidance has just been released in Australia to consider changes in catchment losses, temporal profiles and Probable Maximum Precipitation values [24], but these examples represent minor progress given the weight of evidence and urgency for their development.

The challenge for scientists and practitioners alike is to share a common understanding of the issues involved. To make progress, we need to ensure that the right research questions are being asked, and that appropriate levels of governance are in place to ensure that research outcomes can be translated into improving industry practice. It is thus incumbent on universities to work with industry to ensure that training and research are based on a combination of the best available science and an understanding of best industry practice.

## 6. Conclusions

The explication presented here focuses on the challenges involved in using design-event procedures in a variable and changing climate, and specifically addresses the core assumption that is common to all such methods that the exceedance probability of the derived flood is the same as its causative rainfall. An example based on inductive reasoning was used to illustrate the point that unless an explicit account is given to the joint probabilities involved, then a deterministic design-event method is unlikely to preserve the exceedance probabilities in the transformation of rainfall quantile to flood peak. It would thus be safer to view the fundamental concept of probability-neutrality inherent in deterministic methods as an act of faith rather than a defensible assumption, particularly in applications where there are no data available for independent verification. Existing deterministic procedures can be implemented in stochastic frameworks, and such approaches provide the means to adequately account for both natural variability and the effects of global warming. While continuous simulation approaches are well suited to solving the joint probability issues involved in some practical problems, in many cases, their use introduces other problems that are better solved by event-based approaches.

There are two competing challenges to developing new methods to advance our capability: on the one hand, to facilitate adoption and broad uptake by both decision-makers and service providers, any improvements will need to leverage the value of existing design practice and design datasets; but on the other, we need to recognize that a paradigm shift is required to address the fundamental limitations of traditional methods. Identifying the research gaps relevant to this problem is somewhat of a challenge for two reasons: first, the current procedures used to characterize flood risk vary over jurisdictional scales that span national and local administrative boundaries; and second, the methods and the design information used in their application are generally documented in grey literature that are not captured by academic bibliographic databases.

A key conclusion drawn here is that, to date, there has been a lack of research focus on developing scientifically defensible approaches and tools suited for use by practitioners. We need to take appropriate account of the joint probabilities involved in flood generation and in the key hydroclimatic factors subject to change under global warming. Traditional design-event methods need to be embedded within a stochastic framework to provide the means to take account of both aleatory and epistemic sources of uncertainties. Happily, such a paradigm shift represents a straightforward adoption pathway for practitioners, as the calibration and application of the required frameworks are based directly on traditional design-event procedures. The research required to develop these new approaches can take good advantage of existing research outcomes and design tools, and represents a modest level investment that will greatly increase the defensibility of flood estimates.

## Data Availability

Supplementary material is available online [75].
